# A Dark Target Detection Method Based on the Adjacency Effect: A Case Study on Crack Detection

**DOI:** 10.3390/s19122829

**Published:** 2019-06-25

**Authors:** Li Yu, Yugang Tian, Wei Wu

**Affiliations:** 1 School of Geography and Information Engineering, China University of Geosciences, Wuhan 430074, China; yulityrcug@163.com; 2 China Energy Engineering Group Guangdong Electric Power Design Institute Company Limited, Guangzhou 510700, China; wuwei_cug@163.com

**Keywords:** dark target detection, the adjacency effect, low-high threshold strategy, Gaussian distribution

## Abstract

Dark target detection is important for engineering applications but the existing methods do not consider the imaging environment of dark targets, such as the adjacency effect. The adjacency effect will affect the quantitative applications of remote sensing, especially for high contrast images and images with ever-increasing resolution. Further, most studies have focused on how to eliminate the adjacency effect and there is almost no research about the application of the adjacency effect. However, the adjacency effect leads to some unique characteristics for the dark target surrounded by a bright background. This paper utilizes these characteristics to assist in the detection of the dark object, and the low-high threshold detection strategy and the adaptive threshold selection method under the assumption of Gaussian distribution are designed. Meanwhile, preliminary case experiments are carried out on the crack detection of concrete slope protection. Finally, the experiment results show that it is feasible to utilize the adjacency effect for dark target detection.

## 1. Introduction

Dark target detection based on high resolution and high contrast images, such as crack detection and shadow detection, is important for engineering applications. The existing detection methods can be divided into three types. The first is the threshold method [1,2,3,4,5,6]; the OTSU method [7] and the iterative threshold method [8] are common threshold methods. The threshold method is simple but sensitive to noise. The second is the classification-based target detection algorithm and this method makes full use of spectral and texture information. It includes traditional methods such as K-means [9], support vector machine (SVM) [10]. Meanwhile, machine learning technology [11,12,13,14] has greatly progressed in recent years and has been introduced into target detection. However, labeled data are costly and time-consuming to obtain. The third type of method is connected component analysis [15,16,17], such as the percolation model [18,19] and stroke width transform (SWT) algorithm [20], which mainly utilizes the relationship between the target and its neighboring regions. However, these methods do not consider the imaging environment of a dark target, such as the adjacency effect.

The adjacency effect is also known as cross radiance. This effect is a physical phenomenon caused by atmospheric crosstalk between fields of different surface reflectance. Under the assumption that atmospheric interference has been eliminated, due to the adjacency effect, the surface-leaving radiance from areas adjacent to the target pixel enhances the signal received at the sensor and cause the contrast degradation, blurring of sharp boundaries, reduced resolution, and the difficulty of atmospheric remote sensing [21,22,23]. Further, the adjacency effect will be more important for higher spatial resolution data than Moderate Resolution Imaging Spectroradiometer(MODIS) with 250–500 m pixels and some studies also state that the effect can be observed in high spatial resolution (<100 m) imagery [24,25,26,27]. Meanwhile, this effect also has the largest impact on high-contrast scenes where bright surfaces, such as land or clouds, are adjacent to dark surfaces such as water [28,29,30,31,32]. 

Most researchers have focused on how to model and characterize the adjacency effect and proposed parameterizations of the atmospheric point spread function (PSF), and these were used to correct the adjacency effect [33,34,35,36]. The adjacency effect could be described as the convolution between the radiance field and PSF. If the PSF is known, the adjacent effect could be removed by the deconvolution algorithm. So the core problem with respect to the adjacent effect is to solve the atmospheric PSF [37,38]. There are two main methods for obtaining the PSF. The first is the implementation of the radiation transfer equation by parameterizing various atmospheric and observational conditions [39,40,41,42,43]. The second is the Monte Carlo simulation [44,45,46,47]. Further, there are also some statistical methods, such as the bilinear mixing model or dark spectrum fitting, to simulate or eliminate the adjacency effect [48,49,50]. All in all, current research focused on how to remove the adjacency effect poses the question of whether it possible to utilize it instead. There is almost no research in this area, so this paper has tried to utilize the adjacency effect. Combined with the correspondence between the dark target’s location and its intensity due to the adjacent effect, a low-high threshold strategy is proposed and the strategy is applied in a simple high resolution and high contrast crack detection scene, namely the expansion joints on concrete slope protection. Furthermore, the canny-morphology method and SWT algorithm are used to compare with the proposed method.

## 2. Materials and Methods

### 2.1. Characteristics of the Adjacency Effect

**Figure 1:**
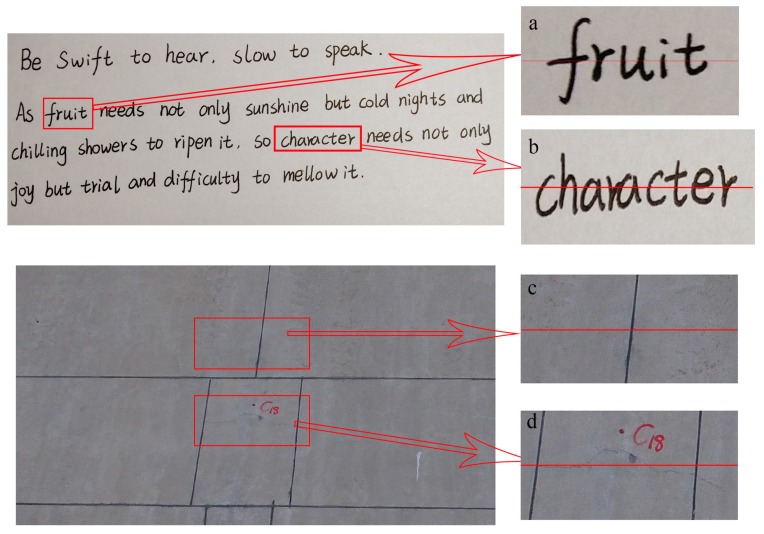
Two typical high-contrast images. And (**a**–**d**) are four example parts of the text image and the crack image.

**Figure 2:**
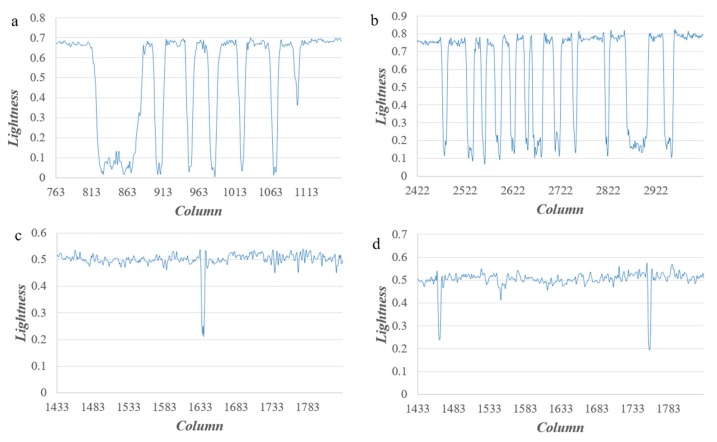
Intensity (Lightness) profiles. And (**a**) is the intensity profile along the line in Figure 1a. (**b**) is the intensity profile along the line in Figure 1b. (**c**) is the intensity profile along the line in Figure 1c. (**d**) is the intensity profile along the line in Figure 1d.

For the high-resolution and high-contrast images where the dark target is surrounded by a bright background, the reflectance of the target pixel contains the contribution of the scattering of background pixels, namely the adjacency effect. Furthermore, the brighter the background pixel is, the more obvious the adjacency effect [51]. The adjacency effect is related to the distance [52] and the further away from a target pixels, the less obvious the adjacency effect is. All in all, the adjacency effect is dependent on the environment [53,54]. There is a correspondence between the dark target’s location and its intensity. Two typical high-contrast images, a text image and a crack image, are selected to display the correspondence as shown in Figure 1. Four parts of them (Figure 1a–d) are selected to show the details, and the intensity (lightness) profiles are obtained along the lines in each part, as shown in Figure 2. Combined with Figure 1 and Figure 2, the intensity value of characters and cracks vary because of the adjacency effect. Further, the lightness profiles show that pixels of characters or cracks at the middle part have a lower intensity value whereas pixels at the edge have a higher intensity value, i.e., there is a correspondence between the dark target’s location and its intensity value due to the adjacency effect.

**Figure 3:**
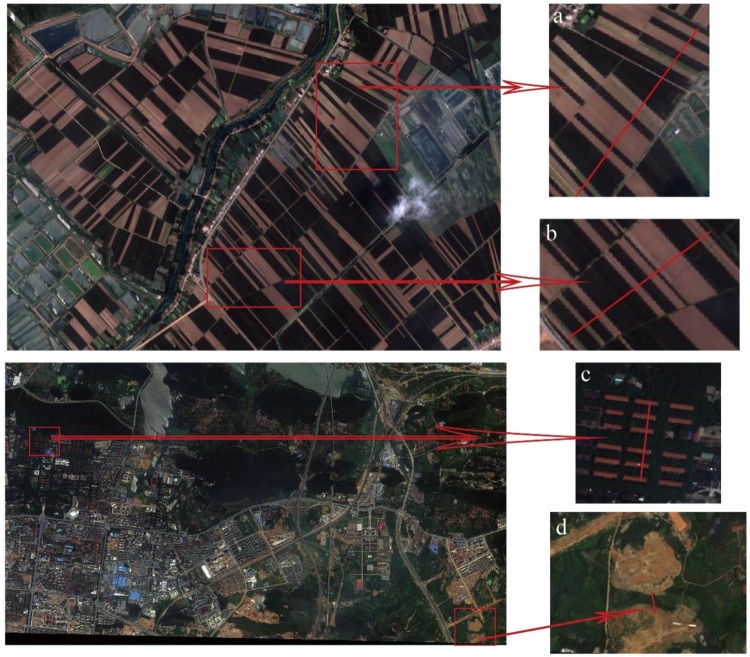
High-contrast scenarios in GF-2 and Worldview-2 images. And (**a**–**d**) are four example parts of the GF-2 image and the Worldview-2 image.

**Figure 4:**
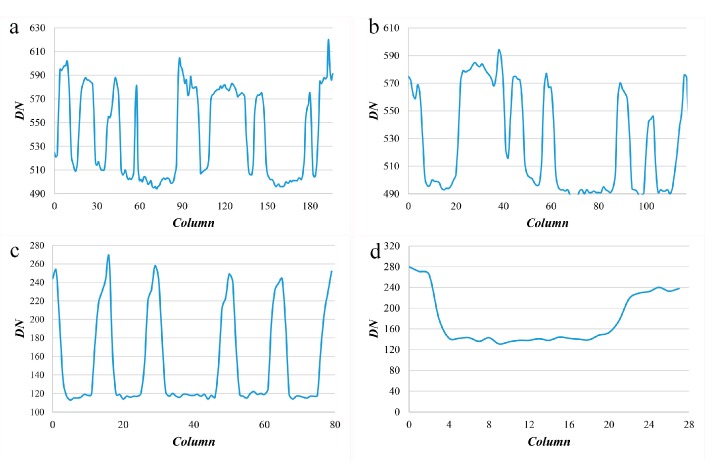
Intensity (Lightness) profiles (only red band is displayed). And (**a**) is the intensity profile along the line in Figure 3a. (**b**) is the intensity profile along the line in Figure 3b. (**c**) is the intensity profile along the line in Figure 3c. (**d**) is the intensity profile along the line in Figure 3d.

Regardless of atmospheric thickness, the correspondence between the dark target’s location and its intensity value is mainly due to the adjacency effect that still exists, as shown in Figure 3 and Figure 4. Two images of GF-2 and Worldview-2 that have not undergone atmospheric correction are selected and four high-contrast parts of them (Figure 3a–d) are selected to show the details. The intensity (lightness) profiles in the red band are obtained along the lines in each part, as shown in Figure 4. 

**Figure 5:**
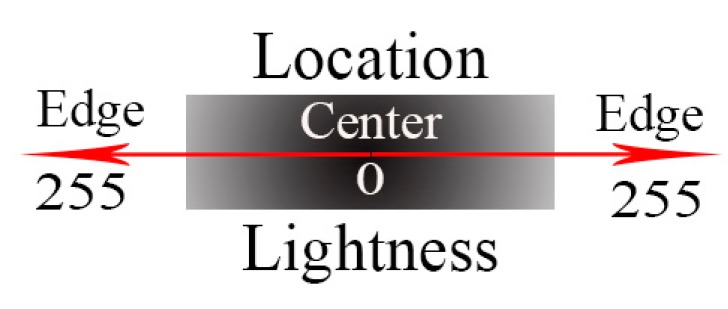
Concept map of the correspondence between the dark target’s location and its intensity value.

Thus, the following conceptual map exists, as shown in Figure 5.

### 2.2. Low-High Threshold Detection Strategy

**Figure 6:**
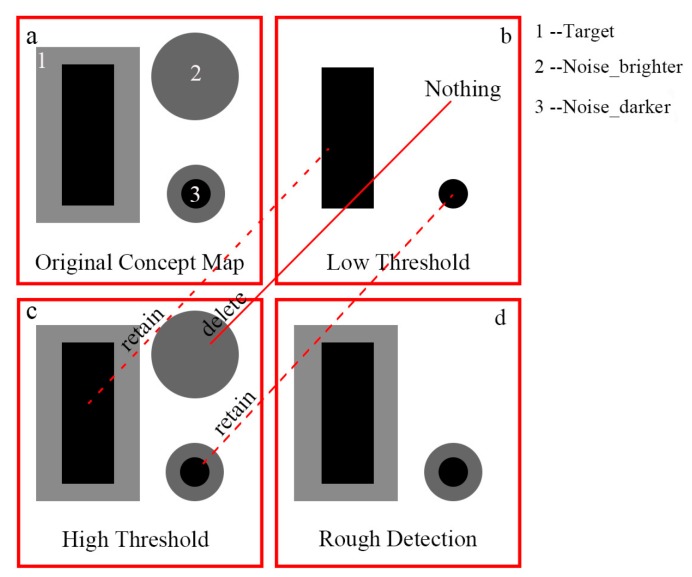
Concept map of Low-High threshold detection strategy. And (**a**) is the conceptual map consists of noise, dark target and bright background. (**b**) is the conceptual map of detected result using a low threshold. (**c**) is the conceptual map of detected result using a high threshold. (**d**) is the conceptual map of rough detection result using the low-high threshold detection strategy.

This paper attempts to utilize the features described in Section 2.1 to detect a dark target surrounded by a bright background in the high contrast and high-resolution image, for which threshold segmentation is a common method. Given the rule displayed in Figure 5, when a small threshold is used, the middle parts of the dark target are detected. As the threshold increases, the edge parts of the dark target are detected gradually until the occurrence of over-extraction. Therefore, this paper proposed a detection strategy to combine under-extraction and over-extraction. First, a low threshold is used to locate the dark target and the result (denoted as $R_{min}$) contains little noise and the middle parts of the dark target. Second, a high threshold is used to detect the complete dark target, however, the result (denoted as $R_{max}$) has considerable noise. Third, if an intersection occurs between $R_{min}$ and the separate unit included in $R_{max}$, then the separate unit is retained; otherwise, it is deleted until all separate units included in $R_{max}$ are traversed. The concept map is illustrated in Figure 6.

**Figure 7:**
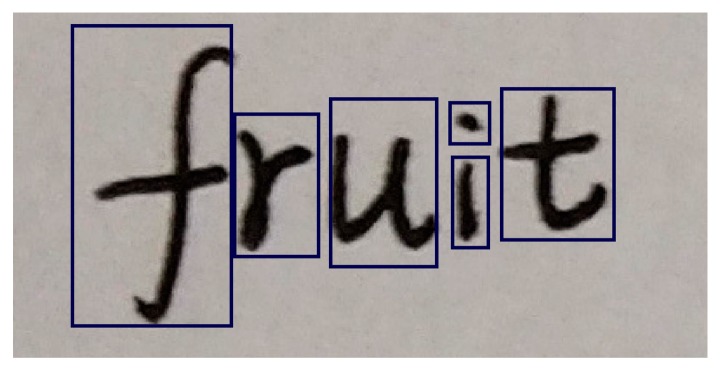
The definition of separate unit.

The detected result contains many parts, every part is a separate unit. For example, Figure 7 contains six separate units, and the rectangular boxes are used to identify them.

### 2.3. Low-High Threshold Selection

#### 2.3.1. The Characteristic of Gaussian Probability Density Function

The Gaussian probability density function is often used as the distribution hypothesis for the statistical model of images; therefore, this paper introduces the Gaussian distribution into the selection of high and low thresholds. The Gaussian probability density function is:

(1) $$f\left(x\right)=\frac{1}{\sqrt{2\pi }\sigma }e^{\left(-\frac{{\left(x-\mu \right)}^{2}}{2\sigma^{2}}\right)}$$

where μ and σ are mean and variance. The first derivative represents the change rate of f(x) along the increasing direction of x, the first derivative equation of f(x) is:

(2) $$f^{\prime }\left(x\right)=-\frac{x-\mu }{\sqrt{2\pi }\sigma^{3}}e^{-\;\frac{{\left(x-\mu \right)}^{2}}{2\sigma^{2}}}$$

Given the derivative test for extremum, the function $f^{\prime }\left(\;x\right)$ takes the extremum at the root of the equation:

(3a) $$f^{\prime \prime }\left(x\right)=0$$

Namely,

(3b) $$f^{\prime \prime }\left(x\right)=-\frac{1}{\sqrt{2\pi }\sigma^{3}}\left(1-\frac{{\left(x-\mu \right)}^{2}}{\sigma^{2}}\right)e^{-\;\frac{{\left(x-\mu \right)}^{2}}{2\sigma^{2}}}=0$$

and the two roots are:

(3c) x=μ±σ

**Figure 8:**
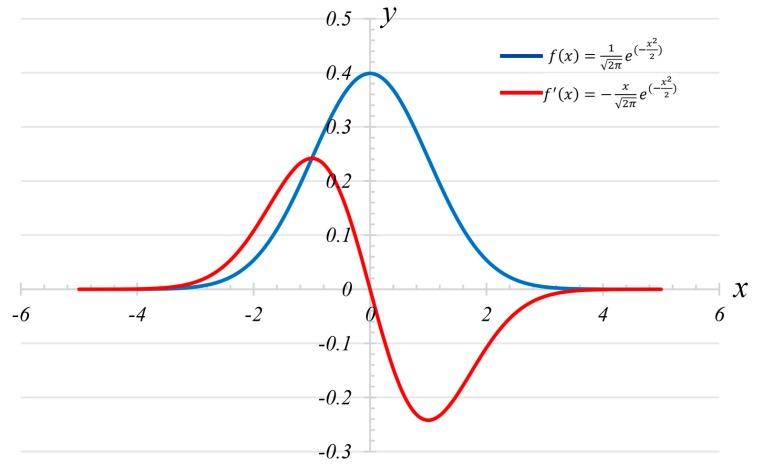
The curve shape of function Gaussian probability density function and its first derivative formula function.

μ and σ only affect the position and width of the curve of the function f(x) and $f^{\prime }\left(x\right)$, and they have no effect on the shape of the curve (bell-shaped symmetrical curve). Therefore, the case where μ is 0 and σ is 1 is used to describe the curve shape of Gaussian probability density function f(x) and its first derivative formula function $f^{\prime }\left(x\right)$, as shown in Figure 8.

According to the curve of $f^{\prime }\left(x\right)$ in Figure 8 and the roots displayed in Equation (3c), the function $f^{\prime }\left(x\right)$ takes the maximum value at point x=μ−σ, where function f(x) has the maximum growth rate. Further, combined with the curve of function f(x) and function $f^{\prime }\left(x\right)$, the function $f^{\prime }\left(x\right)$ is monotonically increasing from negative infinity to μ−σ. Combined with the three-sigma rule, three points are selected as follows:

(4) $$\{\begin{array}{c} f'\left(\mu -\sigma \right)\;=\frac{1}{\sqrt{2\pi }\sigma^{2}}e^{-\frac{1}{2}}\\ f'\left(\mu -2\sigma \right)=\frac{2}{\sqrt{2\pi }\sigma^{2}}e^{-2}\\ f'\left(\mu -3\sigma \right)\;=\frac{3}{\sqrt{2\pi }\sigma^{2}}e^{-\frac{9}{2}} \end{array}$$

Although the value of $f{}^{\prime }\left(x\right)$ varies with σ, the ratio between them is fixed.

(5a) $$\frac{f{}^{\prime }\left(\mu -2\sigma \right)}{f{}^{\prime }\left(\mu -\sigma \right)}=0.44626$$

and

(5b) $$\frac{f{}^{\prime }\left(\mu -3\sigma \right)}{f{}^{\prime }\left(\mu -\sigma \right)}=0.05495\;$$

So even if the mean μ and variance σ are not known, if the fastest growing point ($\mu -\sigma ,f{}^{\prime }\left(\mu -\sigma \right))$ is obtained. By searching for the point forward where the ratio of growth rate to the fastest growing rate is 0.44626, the point ($\mu -2\sigma ,f{}^{\prime }\left(\mu -2\sigma \right))$ is found. Similarly, point ($\mu -3\sigma ,f{}^{\prime }\left(\mu -3\sigma \right)$ can be found.

Furthermore, according to the three-sigma rule, if μ−σ is taken as the threshold, the probability of the numerical distribution in (μ−σ,∞) is:

(6a) 1−(1−0.6827)/2=0.84135

If μ−2σ is taken as the threshold, the probability of the numerical distribution in (μ−2σ,∞) is:

(6b) 1−(1−0.9545)/2=0.97725

If μ−3σ is taken as the threshold, the probability of the numerical distribution in (μ−3σ,∞) is:

(6c) 1−(1−0.9973)/2=0.99865

However, in the actual image, the intensity value is not continuous and the horizontal coordinate interval on the histogram is 1, so the integral of change rates in three intervals are used in place of the three points in Equation (4).

(7) $$\{\begin{array}{c} \int_{\mu -\sigma -1}^{\mu -\sigma }f^{\prime }\left(x\right)dx=f\left(\mu -\sigma \right)-f\left(\mu -\sigma -1\right)=e^{-\frac{1}{2}}-e^{-\frac{{\left(\sigma +1\right)}^{2}}{2\sigma^{2}}}\\ \int_{\mu -2\sigma -1}^{\mu -2\sigma }f^{\prime }\left(x\right)dx=f\left(\mu -2\sigma \right)-f\left(\mu -2\sigma -1\right)=e^{-2}-e^{-\frac{{\left(2\sigma +1\right)}^{2}}{2\sigma^{2}}}\\ \int_{\mu -3\sigma -1}^{\mu -3\sigma }f^{\prime }\left(x\right)dx=f\left(\mu -3\sigma \right)-f\left(\mu -3\sigma -1\right)=e^{-\frac{9}{2}}-e^{-\frac{{\left(3\sigma +1\right)}^{2}}{2\sigma^{2}}} \end{array}$$

The ratio between them is:

(8a) $$\frac{\int_{\mu -2\sigma -1}^{\mu -2\sigma }f^{\prime }\left(x\right)dx}{\int_{\mu -\sigma -1}^{\mu -\sigma }f^{\prime }\left(x\right)dx}=\frac{e^{-2}-e^{-\frac{{\left(2\sigma +1\right)}^{2}}{2\sigma^{2}}}}{e^{-\frac{1}{2}}-e^{-\frac{{\left(\sigma +1\right)}^{2}}{2\sigma^{2}}}}$$

and

(8b) $$\frac{\int_{\mu -3\sigma -1}^{\mu -3\sigma }f^{\prime }\left(x\right)dx}{\int_{\mu -\sigma -1}^{\mu -\sigma }f^{\prime }\left(x\right)dx}=\frac{e^{-\frac{9}{2}}-e^{-\frac{{\left(3\sigma +1\right)}^{2}}{2\sigma^{2}}}}{e^{-\frac{1}{2}}-e^{-\frac{{\left(\sigma +1\right)}^{2}}{2\sigma^{2}}}}$$

The overall illumination image may have an effect on the mean of the image, but it will have little effect on the variance, so the ratio could be calculated by variance in the practical applications.

#### 2.3.2. Low-High Threshold Selection

For the high resolution and high contrast scene where the dark target is surrounded by a bright background, two assumptions are made in the paper: the intensity value of background pixels obeys the Gaussian distribution and the proportion of background pixels is much larger than the target pixels.

The change rate of the histogram can be calculated by the following Equation:

(9) $$C_{i}=\;\frac{Hist_{i+1}-Hist_{i}}{\left(i+1\right)-i}$$

where $Hist_{i}$ is the number of pixels whose intensity value is i on the gray-level histogram and $C_{i}$ is the change rate at the intensity value i.

The proportion of the growth rate to the maximum growth rate is used as the constraint rules to obtain the high threshold as follows:

(10a) $$T_{max}=MAX\left(I\right)$$

and

(10b) $$I\;\mathrm{satisfies}\;\mathrm{rules}:\;\{\begin{array}{c} \frac{C_{i}}{C_{max}}\leq \alpha \\ i<max \end{array}$$

where $\;T_{max}$ is the selected high threshold and MAX(I) is the biggest, namely, the last element of the array I, and I is the candidate array, including all intensity values i that meet the Equation (10b). Meanwhile, max is the intensity value where the biggest growth rate is obtained,$\;C_{max}$ is the biggest growth rate and α is the constraint ratio. 

The meaning of Equation (10) is to search forward from the maximum growth point along the histogram and find the first point where the proportion of the growth rate to the maximum growth rate is greater than α. Combined with the three-sigma rule and the reasoning process in Section 2.3.1, α take $\frac{e^{-2}-e^{-\frac{{\left(2\sigma +1\right)}^{2}}{2\sigma^{2}}}}{e^{-\frac{1}{2}}-e^{-\frac{{\left(\sigma +1\right)}^{2}}{2\sigma^{2}}}}$ or $\frac{e^{-\frac{9}{2}}-e^{-\frac{{\left(3\sigma +1\right)}^{2}}{2\sigma^{2}}}}{e^{-\frac{1}{2}}-e^{-\frac{{\left(\sigma +1\right)}^{2}}{2\sigma^{2}}}}$, and the former is suitable for the high gray-level mixing between the target and the background and the latter is suitable for the low gray-level mixing between the target and the background.

According to the features described in Section 2.1, the middle pixels of the dark target are in the front of the histogram and the edge pixels of dark target are distributed behind the histogram. If the threshold $T_{max}$ detects all the dark target pixels, pixels that locate in the middle of the dark target and account for  a of the total number of the target pixels can be detected. The detection threshold satisfies the following equation:

(11a) $$T_{D_{center}}\left(a\right)=CUM^{-1}\left(a*CUM\left(T_{max}\right)\right)$$

and

(11b) $$a=\frac{N_{D\_center}}{N_{D}}*100\%$$

where CUM( ) is the cumulative distribution function, $CUM^{-1}\left(\;\right)$ is the inverse of the cumulative distribution function. $N_{D\_center}$ is the number of pixels that locate in the center of the dark target, $N_{D}$ is the total number of the dark target pixels, and $T_{D_{center}}$ is the corresponding detection threshold. In this paper, the proportion of a=1/3 is recommended to obtain low threshold. Because only when the width of the dark target is greater than or equal to 3, the corresponding relationship between the dark target’s location and its intensity value due to the adjacency effect could be reflected. So, the maximum value of a is 1/3, and it is selected to ensure that all areas affected by the adjacency effect are detected; thus, the selected low threshold is:

(12) $$T_{min}=CUM^{-1}\left(\frac{1}{3}*CUM\left(T_{max}\right)\right)$$

where CUM( ) is the cumulative distribution function, $CUM^{-1}\left(\;\right)$ is the inverse of the cumulative distribution function. $T_{max}$ and $T_{min}$ are the selected high threshold and low threshold respectively.

#### 2.3.3. Spatial Resolution of Data 

When the proposed method is applied, there are two requirements for image resolution. First, the value of resolution should less than 250 m or 100 m, because the adjacency effect will be more important for higher spatial resolution data than MODIS with 250–500 m resolution and some studies also state the effect can be observed in high spatial resolution (<100 m) imagery. Second, only when the width of the target reaches the three pixels in the images, the difference brought by the adjacency effect between the edge and middle pixels can be reflected, and thus the proposed adaptive threshold selection method can be applied. So, the ratio between the shortest width of the dark target surrounded by a bright background and image resolution should be no less than 3. 

Therefore, the spatial resolution of data should satisfy Equation (13):

(13) $$\{\begin{array}{c} SR\leq 100\;m\;or\;250\;m\\ SR\leq TW_{min}/3 \end{array}$$

where SR is the spatial resolution of data and $TW_{min}$ is the shortest width of the dark target surrounded by a bright background.

## 3. An Application in Crack (Expansion Joint) Detection

### 3.1. Data Selection and Introduction

**Figure 9:**
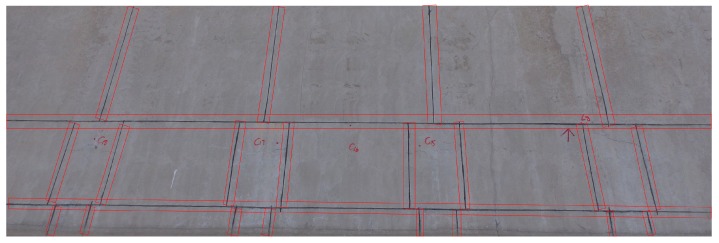
Regular expansion joints on the surface of concrete slope protection.

The expansion joint is a kind of artificial cutting crack. It is designed to safely absorb the temperature-induced expansion and contraction of concrete materials, absorb vibration, or allow movement due to ground settlement or earthquakes. Expansion joints have strict construction specifications, and their design and construction refer to the “Technical Specification for Inspection of Concrete Defects by Ultrasonic Method” which stipulates that a vertical and horizontal expansion joint should be set every 3–5 m, and the width should be 2–3 cm. The expansion joints are included in the red rectangular frame in Figure 9. In summary, the expansion joints have a relatively uniform width and grayscale. So, for every expansion joint in an image, the degree affected by the adjacency effect is similar. Therefore, it is chosen as the research data.

**Figure 10:**
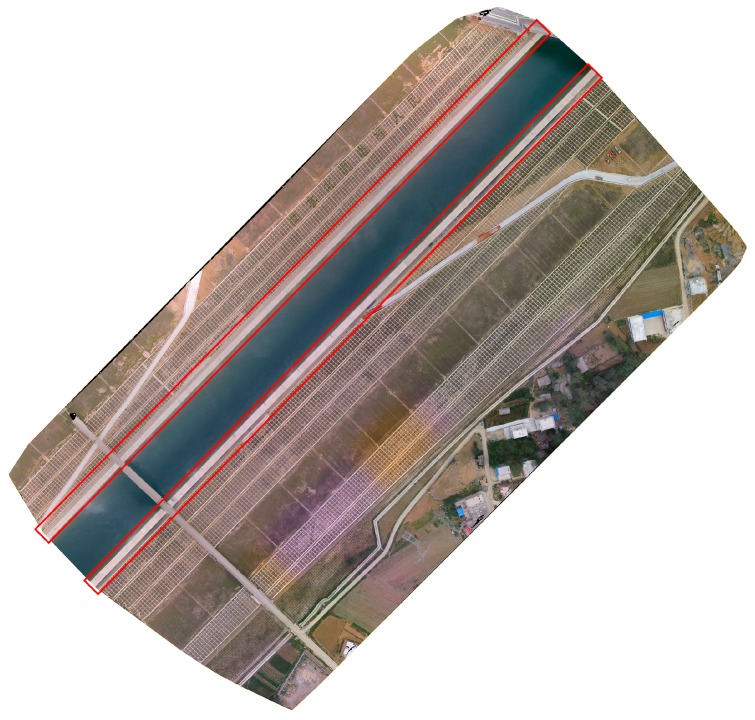
Overall concrete slope protection project.

A total of 18 UAV high resolution and high contrast images in a concrete slope protection project are used in this study. They are RGB images of 4000 × 3000 pixels and the ground sampling distance is about 5 mm. Given Section 2.3.3, the resolution of images should be higher than 2/3 and the UAV images can satisfy the resolution requirement. However, the images are resized to remove water and trees at the side of the slope projection before the experiments. The overall concrete slope protection project is shown in Figure 10 and the test areas are included in the red rectangular frame.

### 3.2. Result and Analysis

The data satisfies the assumption described in Section 2.3.2. The variance of background is 3.495 by simple statistics and there is a low gray-level mixing of the target and background. According to Equation (8b), the constraint ratio is:

(14) $$\frac{e^{-\frac{9}{2}}-e^{-\frac{{\left(3\sigma +1\right)}^{2}}{2\sigma^{2}}}}{e^{-\frac{1}{2}}-e^{-\frac{{\left(\sigma +1\right)}^{2}}{2\sigma^{2}}}}=0.346$$

Therefore, the threshold for the expansion joint detection of a single image is:

(15a) $$I_{e}\;\mathrm{satisfies}\;\mathrm{rules}:\;\{\begin{array}{c} \frac{C_{i}}{C_{max}}\leq 0.346\\ i<max \end{array}$$

(15b) $$T_{emax}=\;I_{e}\left[last\right]$$

and

(15c) $$T_{emin}=CUM^{-1}\left(\frac{1}{3}*CUM\left(T_{emax}\right)\right)$$

where $I_{e}$ is the candidate array including all intensity values i that meet the Equation (15a), $T_{emax}$ and $T_{emin}$ are the high and low threshold selected to detect expansion joints respectively.

The rough detection results are obtained by $T_{emax}$ and $T_{emin}$ according to the strategy described in Section 2.2. The rough detection results consist of expansion joints and some noise which has the same gray-level distribution with expansion joints, however, there are morphological and geometric differences between them. So, the different constraint conditions are set up to remove noise and achieve the accurate detection of the expansion joints.

The morphological characteristic is used to remove other noise. Because expansion joints exhibit linear morphological characteristics, and the shape of other noise is close to circular. Thus, the circularity $F_{c}$ is used as a constraint condition to remove the other noise, and it is expressed by the following equation:

(16) $$F_{c}=\frac{4C_{count}}{\pi C^{2}{}_{max}}$$

where $C_{count}$ is the number of pixels in every separate unit of detected results, and $C_{max}$ is the maximum length of the separate unit. Based on the equation above, the $\;F_{c}$ value ranges from 0 to 1. Furthermore, the $F_{c}$ value of an image is close to 1 when the shape of the separate unit is nearly circular, and the $F_{c}$ value of the image is close to 0 when the shape of the separate unit is linear. After the trial-and-error experiment, when the value of $\;F_{c}$ is greater than 0.18, the separate unit is divided into noise, otherwise, it is divided into the expansion joints.

Besides, the geometric characteristic is used to remove other noise. Because the expansion joint is continuous and has a large area while most of noise has a small area, the area constraint is used to remove the noise. After the trial-and-error experiment, when the area of the separate unit is less than 500, the separate unit is divided into noise, otherwise, it is divided into the expansion joints. 

In order to evaluate the detection capacity of the proposed method, the canny-morphology method is selected. Meanwhile, the SWT algorithm is first introduced into this field. Social Media event detection is a major direction of visual event analysis [55,56,57,58] and text attribute is a critical part of semantic visual attributes [59,60], so a text recognition method, the SWT algorithm [61,62,63,64], which is used for text detection comes into being. The algorithm mainly utilizes the uniform width of the stroke and the expansion joints also have a uniform width, so it is selected. Furthermore, the manually drawn sketch is used as ground-truth reference data to evaluate the accuracy of these detection methods. The three evaluation indices are represented as follows:

(17) $$\{\begin{array}{c} Recall=\frac{Area_{Objeict\;\cap \;Objeict{\;}_{m}}}{Area_{Object_{m}}}\\ Precision=\frac{Area_{Objeict\;\cap \;Objeict{\;}_{m}}}{Area_{Object}}\\ F-measure=\frac{2*Recall*Precision}{Recall+Precision} \end{array}$$

where $Area_{Object_{m}}$ denotes the area of the result produced by manually drawing, $Area_{object}$ denotes the area of the detected result produced by the detection method, and $Area_{Objeict\;\cap \;Objeict{\;}_{m}}$ denotes the area of the product set between $Area_{Object_{m}}$ and $Area_{object}$. Based on Equation (17), the Recall value and Precision values range from 0 to 1. Meanwhile, the F-measure combines the results of Precision and  Recall, and the higher the F-measure is, the more accurate the detected result is.

**Figure 11:**
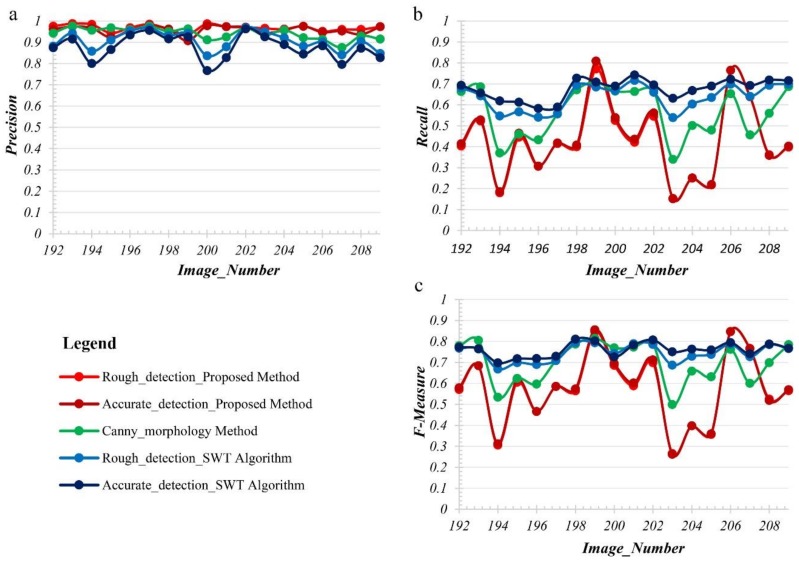
Detection accuracy for expansion joints of all images. And (**a**) is the Precision curves using the proposed method, the canny_morphology method, and the SWT Algorithm. (**b**) is the Recall curves using the proposed method, the canny_morphology method, and the SWT Algorithm. (**c**) is the F-measure curves of using the proposed method, the canny_morphology method, and the SWT Algorithm.

The comparison results of the three methods are displayed in Table 1 and Figure 11. The accuracy between rough detection and accurate detection is not much different, as shown in Figure 11. It shows that the rough detection method itself is effective and accurate detection only further improves the accuracy of the method by increasing certain constraints.

The detectability of the three methods is quantitatively assessed using the evaluation indices described in Equation (17). The higher the value of precision is, the higher the completeness of the detection result is. Combined Figure 11 and Table 1, the final Precision of the three methods is high, and the proposed method has the highest  Precision. The mean Precision of the proposed method, canny-morphology method and SWT algorithm is 95.76%, 94.09%, 87.67% respectively. Meanwhile, the higher the value of Recall is, the less noise is included in the detection results. However, the Recall of the three methods is much lower than their precision. The mean Recall of them is 43.69%, 56.79%, 67.55% respectively. There are many edge portions of expansion joints which are artificially divided into the background pixels, so the area of reference data (manually drawn sketches) is smaller than the real expansion joints, which leads to the overall low Recall. Further, the mean Recall of the proposed method is lower than the other two methods and the reason for this is the interference of dark noise connected to the expansion joints (denoted as D-c-E). Further, the D-c-E also reduces the Recall of canny-morphology method and SWT algorithm; however, due to the constraint of the convolution kernel radius and the width of the stroke, only a small amount of noise pixels are detected by mistake or a small amount of expansion joint pixels are leaked, especially for those large D-c-E. So, the D-c-E has less influence on the two methods than the proposed method. However, if there is no the D-c-E, the Recall of the proposed method is higher than the comparison method, such as image 8 and image 15, the Recall of the proposed method is 80.90% and 76.50%, respectively, as shown in Table 1.

**Figure 12:**
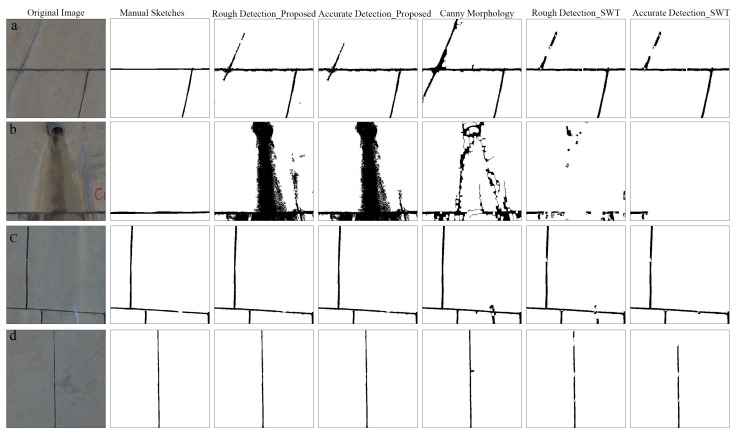
(**a**–**d**) are four typical partial examples of expansion joint detection. Columns from left to right are the original images, manually drawn sketches, rough detection results using the proposed method, accurate detection results using the proposed method, detection results using the canny-morphology method, rough detection results using the SWT algorithm and accurate detection results using the SWT algorithm.

Four typical partial examples of expansion joint detection are shown in Figure 12. 

Figure 12a is a partial image including the D-c-E which has a width and intensity value close to the expansion joint. It is difficult to remove such interference for the three methods. The canny-morphology method is most affected because the morphological operation connects the discontinuous parts in D-c-E.

Figure 12b is a partial image including the D-c-E which has a large width and area; further, its intensity value is close to the expansion joint. For such noise, the proposed method is most affected because the D-c-E is detected entirely using the proposed method, which makes Recall drop drastically. However, due to the limitation of the convolution kernel radius, only the edge part of this noise is detected incorrectly using the canny-morphology method, which makes Recall drop little. Meanwhile, due to the constraint of the width of the stroke, the part of expansion joints connected to the dark noise is deleted incorrectly using SWT algorithm, which makes Recall rise a little. 

Figure 12c is a partial image including the white noise which is connected to the expansion joint and has a width close to the expansion joints. Such noise can be easily removed using the proposed method. However, such noise will be mis-detected using the canny-morphology method because the edges of white noise are detected and retained. For the SWT algorithm, the part of expansion joints connected to the noise is deleted due to the constraint of the width of the stroke, and the residual part of such noise might be removed in the process of accurate detection. 

Figure 12d is a partial image including expansion joint with uneven width. Both the proposed method and the canny-morphology method perform well in this situation. However, due to the constraint of the width of the stroke, parts of expansion joints where the width of the expansion joints is too thin and too thick are deleted incorrectly using the SWT algorithm. 

The three methods are realized using MATLAB R2014a on the operating environment, Windows 7, and a processor, Intel(R) Core (TM) i-7400 at 3.00 GHz 3.00 GHz.

The computational complexity of the three methods is described from two aspects of pace and time. The space complexity of these methods is related to the image size. The main time consumption of the proposed method is shown on the process of low-high threshold detection; however, the algorithm has been optimized by the matrix operation and the conditional statement. The time consumption of the canny-morphology method is mainly spent on the process of edge detection. Meanwhile, the traversal calculation of stroke leads to the long-running time of the SWT algorithm and increases with the size of images.

The running time of the three methods is listed in Table 2. The average running time is 1.6090 s, 2.8076 s, and 4.4627 s separately. Therefore, the proposed method has the best performance although its running time varies with images that have different numbers of separate units. The Canny-morphology method has stable performance, and its running time of the Canny-morphology method is fewer changes with the images. The SWT has the worst performance, and its running time varies with image sizes.

## 4. Conclusions and Discussion

In this paper, a dark target detection method based on the adjacency effect is proposed, and a typical simple scene uniformly affected by the adjacency effect is selected for application experiments. By comparing with the canny-morphology method and the SWT algorithm, it is found that the proposed method can realize the complete detection of expansion joints and it is feasible to utilize the adjacency effect for dark target detection. Furthermore, because only RGB images are needed, the scope of application of the study is wide. However, although the dark noise connected to the dark target is a general problem of various detection methods, the proposed method is more affected. Besides, the application of the adjacency effect in complex scenarios and the detection effect with different resolution remains to be further explored.

## Figures and Tables

**Table 1:** Detection accuracy for expansion joints of all the images. Precision_P, Precision_C, and Precision_C are precision of the proposed method, canny-morphology method, and SWT algorithm respectively. Similarly, Recall_P, Recall_C, and Recall_C are recall of the proposed method, canny-morphology method, and SWT algorithm respectively.

*Number*	*Precision_P*	*Recall_P*	*Precision_C*	*Recall_C*	*Precision_S*	*Recall_S*
1	0.9612	0.4142	0.9426	0.6639	0.8744	0.6932
2	0.9740	0.5282	0.9762	0.6860	0.9147	0.6571
3	0.9667	0.1859	0.9563	0.3704	0.8000	0.6191
4	0.9225	0.4647	0.9676	0.4599	0.8652	0.6125
5	0.9571	0.3081	0.9587	0.4330	0.9347	0.5834
6	0.9787	0.4179	0.9759	0.5560	0.9554	0.5899
7	0.9614	0.4085	0.9504	0.6718	0.9160	0.7274
8	0.9073	0.8090	0.9624	0.7075	0.9253	0.7087
9	0.9777	0.5395	0.9114	0.6656	0.7669	0.6903
10	0.9733	0.4360	0.9249	0.6641	0.8279	0.7424
11	0.9710	0.5619	0.9667	0.6641	0.9627	0.6951
12	0.9434	0.1522	0.9328	0.3403	0.9256	0.6320
13	0.9567	0.2515	0.9575	0.5020	0.8892	0.6682
14	0.9754	0.2203	0.9209	0.4805	0.8444	0.6901
15	0.9471	0.7650	0.9136	0.6535	0.8832	0.7222
16	0.9533	0.6394	0.8754	0.4567	0.7961	0.6927
17	0.9370	0.3587	0.9291	0.5597	0.8715	0.7184
18	0.9732	0.4025	0.9146	0.6874	0.8273	0.7158

**Table 2:** Running time for expansion joints of all the images using three method.

*Number*	*RT-Proposed Method(s)*	*RT-Candy-Morphology(s)*	*RT-SWT(s)*
1	2.1285	2.9224	4.2328
2	1.1376	2.0462	3.6519
3	3.8280	3.4582	4.9786
4	1.0969	2.7031	4.3437
5	1.2345	2.8442	4.1065
6	1.2428	2.6918	3.9110
7	1.9997	2.7717	3.9742
8	0.8414	2.4180	4.0313
9	1.6320	3.0660	4.8097
10	2.7472	3.4834	5.3840
11	1.2736	2.6746	5.5774
12	1.2082	2.8016	4.2024
13	0.9160	2.2509	4.0607
14	1.4074	2.9806	4.9223
15	0.8659	2.9569	4.4141
16	1.0288	3.0996	4.7188
17	1.8465	2.6030	4.0301
18	2.5270	2.7639	4.9794
